# Integrating Telerehabilitation into the Prehabilitation and Rehabilitation Pathway in Colorectal Cancer: A Case Series

**DOI:** 10.3390/reports8020060

**Published:** 2025-04-30

**Authors:** Jose Manuel Burgos-Bragado, Natalia Brandín-de la Cruz, Beatriz Carpallo-Porcar, Juan Luis Blas-Laina, Sandra Calvo, Carolina Jiménez-Sánchez

**Affiliations:** 1 Department of Physical Therapy, Faculty of Health Sciences, Universidad San Jorge, 50830 Villanueva de Gállego, Zaragoza, Spain; jmburgos@usj.es (J.M.B.-B.); nbrandin@usj.es (N.B.-d.l.C.); bcarpallo@usj.es (B.C.-P.); cjimenez@usj.es (C.J.-S.); 2 Hospital Royo Villanova, 50015 Zaragoza, Spain; jlblas@salud.aragon.es; 3 Department of Physiatry and Nursing, Faculty of Health Sciences, University of Zaragoza, 50009 Zaragoza, Spain; 4 IIS Aragón, 50009 Zaragoza, Spain

**Keywords:** colorectal cancer, telerehabilitation, prehabilitation, rehabilitation, physiotherapy

## Abstract

**Background and Clinical Significance:** Colorectal cancer (CRC) remains a global health challenge with significant postoperative complications and functional declines. Telerehabilitation offers an accessible alternative to improve preoperative physical condition and postoperative recovery. **Case Presentation:** Five CRC patients scheduled for laparoscopic surgery participated in an asynchronous telerehabilitation multimodal program, including two weeks of prehabilitation and four weeks of postoperative rehabilitation. Delivered via a digital platform with remote physiotherapist support, the intervention improved functional capacity and muscle strength preoperatively, with partial recovery noted post-surgery. **Conclusions:** Integrating telerehabilitation into the CRC surgical pathway is feasible and may enhance functional outcomes and quality of life. Further studies are required to confirm these preliminary findings.

## 1. Introduction and Clinical Significance

Colorectal cancer (CRC) remains a significant global public health challenge due to its high incidence, substantial mortality rate, and considerable healthcare burden. According to the World Health Organization (WHO), CRC is the third most commonly diagnosed cancer worldwide, accounting for approximately 10% of all cancer cases, and remains the second leading cause of cancer-related mortality [1].

Preventive strategies for CRC primarily target modifiable lifestyle factors, including smoking, alcohol consumption, sedentary behavior, obesity and poor dietary habits. In addition, promoting protective behaviors such as regular physical activity and a balanced diet play a crucial role in reducing CRC risk [2]. Screening programs serve as a key component of secondary prevention, enabling early detection and contributing to lower incidence and mortality rates [3].

Despite significant advances in cancer therapies that have improved survival rates and reduced local recurrence in CRC [4], functional outcomes have not improved at the same pace. Surgical resection, the main approach for CRC, is often associated with significant postoperative complications, prolonged recovery and reduction in functional capacity and quality of life (QoL) [5,6]. These postoperative complications often result in loss of independence and increased healthcare use and costs. In addition, preoperative factors such as fatigue, muscle weakness, malnutrition and reduced physical capacity have been associated with higher complication rates, and delayed recovery [7,8]. To address these issues, integrating structured rehabilitation programs before (prehabilitation) and after surgery is essential to optimize functional recovery, reduce postoperative complications, and improve long-term outcomes [9].

Exercise and rehabilitation are fundamental components of comprehensive cancer care, as recommended in international guidelines [10,11]. Multimodal prehabilitation, which includes structured physical exercise, nutritional optimization, and therapeutic education, has been shown to significantly improve preoperative functional capacity, reduce postoperative complications, and accelerate recovery in patients undergoing CRC surgery [12,13,14]. Similarly, postoperative exercise interventions are essential for restoring physical function, promoting recovery and significantly improving overall QoL [14,15]. Integrating multimodal prehabilitation into ERAS (Enhanced recovery after surgery) protocols improves clinical outcomes, shortens hospital stays and lowers healthcare costs [16,17,18].

Telerehabilitation has recently emerged as an innovative strategy to improve access to rehabilitation services. By using digital platforms to provide structured exercise programs, educational resources, and remote patient monitoring, telerehabilitation offers greater accessibility, flexibility, and treatment adherence compared to traditional in-person rehabilitation [19,20,21]. This approach is particularly beneficial for patients with mobility limitations or those living in geographically remote areas [22]. In oncology, telerehabilitation has shown significant potential to increase patient engagement, maintain functional recovery and enhance QoL, highlighting its valuable role in perioperative care and long-term CRC management [23].

As CRC incidence continues to rise, the need for accessible and effective rehabilitation strategies becomes increasingly relevant. While conventional rehabilitation programs have shown benefits, their availability and patient adherence remain challenging. Telerehabilitation presents a flexible and scalable alternative that could help overcome these barriers by providing remote access to structured interventions tailored to patients’ needs.

The aim of this study is to evaluate the effect of an asynchronous multimodal telerehabilitation program on body composition, functional capacity, muscle strength, psychosocial factors and QoL in patients undergoing CRC surgery.

## 2. Case Presentation

This prospective case series describes five CRC patients who participated in a structured telerehabilitation program integrated into a multimodal prehabilitation strategy prior to CRC surgery, followed by a postoperative rehabilitation phase to improve functional recovery. The intervention was delivered asynchronously via a digital platform with remote monitoring.

The study was conducted between June 2024 and February 2025. Patients were recruited at the Department of General and Digestive Surgery of the Royo Villanova Hospital (Zaragoza, Spain). Ethical approval for the study protocol was granted by the Ethics Committee of Aragón under reference PI23/557. All participants provided written informed consent prior to enrolment, in accordance with the ethical standards before participating in the study.

### 2.1. Eligibility Criteria

The inclusion criteria: (1) Adults aged 18 to 80 years. (2) Patients scheduled for elective CRC surgery at the Hospital Royo Villanova. (3) First consultation at the Department of General and Digestive Surgery. (4) Functionally independent individuals able to perform walking and pulmonary function tests. (5) Preoperative classification of I, II or III according to the American Society of Anaesthesiologists (ASA) classification and (6) Willingness to participate in the study and signed informed consent.

The exclusion criteria: (1) Patients over 80 years old. (2) Preoperative ASA classification of IV. (3) Musculoskeletal, inflammatory or other pathological conditions prevent physical exercise. (4) Central and/or peripheral neurological disorders that limit participation in the rehabilitation program. (5) Unstable concomitant cardiac conditions, including arrhythmias, hypertension, angina or other conditions contraindicating moderate-intensity exercise. (6) Diagnosed psychiatric disorders as determined by a psychiatrist. (7) Lack of access to an internet-enabled mobile device or computer at home and (8) Refusal to participate or lack of a signed consent form.

### 2.2. Cases Characteristics

The sample consisted of five adults (three males and two females) with a mean age of 53.6 ± 10.7 years (range: 36–62 years). Participants’ weight ranged from 53.4 kg to 95.6 kg, with heights varying between 164 cm and 191 cm. Body mass index (BMI) assessments revealed that three participants were classified as overweight (BMI: 25.7–29.8 kg/m^2^), while two participants fell into the normal weight category (BMI: 20.6 and 20.8 kg/m^2^).

Educational backgrounds were diverse, including two participants with university degrees, two with secondary education, and one with primary education. All participants were employed during the study period.

The sociodemographic data collected are listed in Table 1.

### 2.3. Procedure

After the recruitment of participants by the head of oncology at Royo Villanova Hospital, the physiotherapist in charge of the assessment visited Royo Villanova Hospital to conduct the initial and final assessments.

In the initial phase, the participants completed the written scales and followed the physical examination. Once the initial assessment was completed, the physiotherapist in charge of the intervention registered each patient in HEFORA.

HEFORA is a free, asynchronous digital platform for therapeutic exercise prescription and patient education that provides individualized instruction on their use. HEFORA enables remote monitoring and is accessible via mobile phone, tablet or computer and offers flexible participation options.

The physiotherapist installed the platform on each participant’s device and guided them through the navigation, self-monitoring features, and communication tools.

After completion of the onboarding, data collection was conducted at five key time points: Time 1 (T1): pre-prehabilitation (baseline assessment); Time 2 (T2): post-prehabilitation (pre-surgery, the day before surgery); Time 3 (T3): pre-rehabilitation (post-surgery—day 21, after suture removal); Time 4 (T4): post-rehabilitation (day 50, after completion of the rehabilitation phase) and Time 5 (T5): follow-up (three months after T4 by telephone assessment).

Throughout the intervention period, participants engaged asynchronously with the physiotherapist via the HEFORA platform. Patients were instructed to self-report exercise completion, perceived exertion (using a 0–10 scale), and any symptoms or adverse events through weekly digital logs. The physiotherapist reviewed these data three times per week and provided personalized feedback through text messages on the platform. In cases of technical issues or low adherence alerts, follow-up communications, either via phone calls or text messages, were initiated to ensure patient safety and maintain program continuity. This asynchronous model facilitated individualized adjustments and continuous monitoring without the need for real-time sessions.

### 2.4. Intervention

The telerehabilitation program was structured into two phases: a two-week prehabilitation and a four-week postoperative rehabilitation period. Both phases adhered to international rehabilitation guidelines and the recommendations of the ERAS protocol [10,11,18,24].

The prehabilitation period was limited to two weeks due to the public healthcare system’s scheduling policies, which define the timeframe prior to elective colorectal cancer surgery. During this phase, the focus was on enhancing physical readiness for surgery through structured aerobic and resistance exercise, complemented by basic nutritional advice and therapeutic education. As part of the ERAS recommendations, participants followed a low-fiber diet in the eight days preceding surgery to reduce intestinal residue and minimize postoperative complications [18].

All program content was delivered asynchronously through the HEFORA digital platform, enabling flexible participation, remote monitoring, and individualized support. The platform provided patients with a personalized interface featuring educational materials, exercise videos, logging tools, and a messaging system for direct communication with the physiotherapist.

The physiotherapy program included **(1) Therapeutic education:** Participants received prerecorded videos and written materials with evidence-based guidance on prehabilitation principles, postoperative self-care, healthy lifestyle habits, and early mobilization strategies aimed at preventing complications and promoting autonomy during recovery. **(2) Respiratory exercises:** A daily respiratory program including diaphragmatic and costal expansion breathing, controlled breathing techniques and incentive spirometry was prescribed to enhance pulmonary function and reduce postoperative respiratory complications. **(3) Aerobic exercise:** Moderate to vigorous aerobic exercise (e.g., brisk walking, stair climbing) totaling 150–300 min per week was recommended with the aim of gradually improving cardiovascular endurance and overall physical condition. Sessions started at 20–30 min and progressed to 45–50 min, 4–5 days per week. **(4) Strength training:** Resistance exercises targeting major muscle groups were performed 3–4 times per week, using bodyweight, resistance bands, or household objects (e.g., water bottles, backpacks). Typical movements included bent-over rows, wall push-ups, arm curls, front and lateral shoulder raises with weights, squats (including wall squats), lunges, hip flexor activations and gluteus maximus and medius strengthening. Training progressed from 2 to 3 sets of 8 repetitions to 3 sets of 15 repetitions. The entire exercise program was individually tailored according to the patient’s baseline assessment and updated weekly based on self-reported exertion and symptoms. Exercise intensity was guided using a modified Borg Scale (rating 3–7) to ensure safety and effectiveness. Adherence was tracked through the HEFORA platform, where patients recorded their activity completion and reasons for missed sessions. The asynchronous messaging system allowed timely adjustments and real-time support as needed, and the program was adapted to each patient’s limitations.

### 2.5. Outcomes

#### 2.5.1. Primary Outcome

Functional capacity was assessed using the six-minute walk test (6MWT), according to the American Thoracic Society (ATS) and the European Respiratory Society (ERS) guidelines [25,26]. The test measures the maximum distance (meters) a patient can walk in six minutes along a standardized 30 m corridor, providing an objective assessment of aerobic endurance, cardiopulmonary function, and overall physical capacity [27].

The 6MWT is widely used in colorectal cancer rehabilitation, as it assesses the patient’s postoperative recovery and functional progress [28]. This test integrates the responses of the cardiovascular, pulmonary, and musculoskeletal systems, making it a reliable indicator of functional performance over time [29].

#### 2.5.2. Secondary Outcomes

##### Body Composition

Body composition was determined using bioelectrical impedance analysis (BIA) with a Tanita BC-601 (InnerScan^®^V, Tokyo, Japan), a validated, non-invasive method for estimating visceral fat percentage, weight, body fat percentage, muscle mass and body water percentage [30]. The BIA method is widely used in clinical and research settings due to its reproducibility and ability to monitor longitudinal changes in body composition [31]. These parameters provide a comprehensive overview of metabolism, muscle condition and fat distribution, all of which are critical for the assessment of nutritional status and functional outcomes in CRC patients.

Waist and hip circumference were also measured according to a standardized anthropometric protocol to assess central adiposity, which is strongly associated with metabolic risk, systemic inflammation, and cancer prognosis [30]. In CRC rehabilitation, these indicators are particularly relevant, as increased visceral fat and sarcopenia have been associated with poorer functional outcomes, higher postoperative complication rates, and delayed recovery [32].

##### Muscle Strength

Muscle strength was assessed using two validated functional tests: the Handgrip Strength Test (HGS) for upper limb strength and the Five-Repetition Sit-to-Stand Test (5R-STS) for lower limb function. Both tests are commonly used in oncologic rehabilitation to evaluate neuromuscular performance, functional independence, and postoperative recovery [33].

Upper limb strength was measured using a hydraulic hand-held dynamometer in a standardized seated position (shoulder aligned, elbow at 90°, wrist in neutral position). Each patient performed three maximal voluntary contractions per hand, held for three seconds, and the highest value was used for analysis. Handgrip strength is a recognized marker of sarcopenia, recovery ability, and surgical outcome, with lower values associated with increased postoperative complications [34].

Lower limb strength was evaluated using the 5R-STS, which required patients to stand up and sit down five times as quickly as possible with their arms crossed over the chest. The total time (in seconds) was recorded, using a standardized chair height (43–47 cm) for consistency. This test is closely related to functional mobility, fall risk and postoperative recovery [35].

##### Psychosocial Factors

Psychosocial adjustment was assessed using two validated, self-administered instruments: the mental adjustment to cancer (MAC) scale and the hospital anxiety and depression scale (HADS).

The MAC scale evaluates patients’ cognitive-behavioral coping styles in response to cancer. It comprises five subscales: Fighting Spirit, Anxious Preoccupation, Fatalism, Helplessness/Hopelessness, and Cognitive Avoidance [36]. Higher scores on Positive Adjustment dimensions (e.g., Fighting Spirit, Positive Orientation to the Illness) indicate active and adaptive coping, which is generally associated with better psychological well-being and better adherence to treatment. In contrast, elevated scores on Negative Adjustment dimensions (e.g., Help-less–Hopelessness, Anxious Preoccupation, Fatalism) suggest maladaptive responses, such as emotional distress, avoidance or resignation, which are associated with poorer QoL and prognosis [36,37].

The hospital anxiety and depression scale (HADS) is a validated instrument for assessing clinically relevant anxiety symptoms (HADS-A) and depression (HADS-D) and is commonly used in oncology to evaluate psychological distress and its impact on QoL and treatment adherence [38]. It comprises 14 items that are evenly divided into two subscales, each of which can be scored from 0 to 3, so that the total subscale scores range from 0 to 21. The generally accepted interpretation thresholds are 0–7 (normal), 8–10 (borderline or mild symptoms) and ≥11 (probable clinical case). In cancer patients, elevated HADS scores have been consistently associated with decreased QoL, increased emotional distress, and decreased adherence to treatment. Accordingly, interpretation of scores should be placed in the context of the patient’s overall clinical profile, and elevated scores should lead to a more comprehensive psychosocial assessment and targeted psychological or emotional support interventions as appropriate [39].

##### Quality of Life EuroQol 5D

Health-related quality of life (HRQoL) was assessed using the EuroQol-5D (EQ-5D) questionnaire, a standardized, self-administered instrument widely used in both clinical practice and research to evaluate overall health status and patient-reported outcomes. Consists of two subscales: the EQ-5D descriptive system and the EQ visual analogue scale (EQ-VAS). The EQ-5D descriptive system includes five core dimensions: mobility, self-care, usual activities, pain/discomfort, and anxiety/depression. Each dimension is scored on five levels (EQ-5D-5L), allowing for a more detailed assessment of functional and psychological well-being [38]. The combination of scores for the five dimensions results in a five-digit code representing the person’s health profile, which is then converted into a utility index (EQ-5D index) ranging from 0.000 (equivalent to death) to 1.000 (perfect health). In addition, the EQ-VAS records the patient’s self-rated health on a scale from 0 (worst imaginable state of health) to 100 (best imaginable state of health), which is a subjective measure of perceived health [40].

In the context of oncology rehabilitation, particularly in the treatment of CCR patients, the EQ-5D is often used to monitor changes in physical functioning, symptom burden and emotional state to provide a comprehensive understanding of the impact of treatment and recovery [41].

### 2.6. Results

Functional Capacity

All participants improved their functional capacity as measured by the 6MWT during the prehabilitation phase, with participant 3 reporting the greatest improvement. In general, all participants exhibited a preoperative increase of 118.8 m (21.3%) from T1 (557.2 ± 47.0 m) to T2 (676.0 ± 33.6 m) (Table 2).

After surgery, a post-surgery decline was observed in all participants between T2 and T3 (127.4 m) (18.8%) (Table 2).

**Figure 1:**
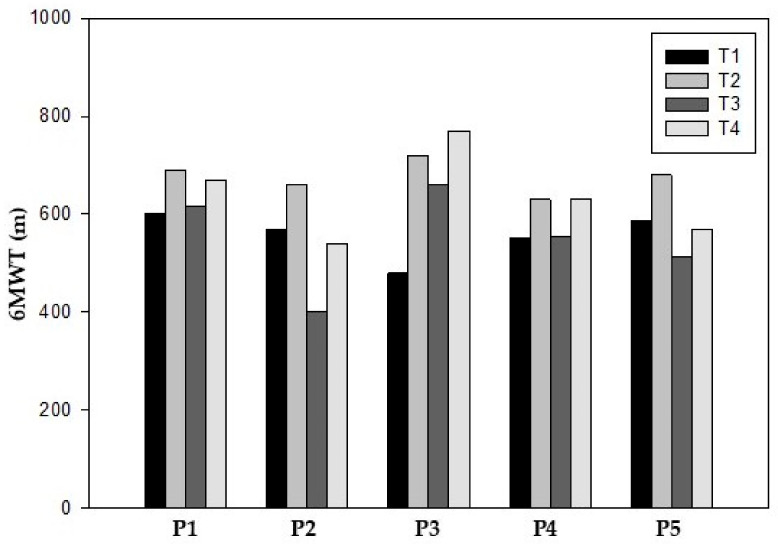
Six-minute walk test.

In the final assessment at T4, all participants improved compared to T3 (Median 54 m), with participant 2 improving the most. In addition, only participants 1, 3 and 4 showed an increase in meters compared to the baseline values (Table 2) (Figure 1).

Body composition

Changes in body composition (Table 3) reflected the combined effects of prehabilitation, surgery, rehabilitation, and dietary reintroduction.

With regard to the waist circumference, all participants except participant 2 reduced their circumference during the prehabilitation phase and after surgery (T2). In post-surgery rehabilitation, waist circumference increased in all participants when comparing T3 and T4. Regarding T4, participants 1, 2, 3 and 5 had lower values with respect to baseline (Table 3).

In terms of hip circumference, the only participant who did not reduce their perimeter was participant 1. T3 and T4 followed a similar trend recovering similar values to baseline, with the exception of participant 2 (Table 3).

In general, all participants presented similar values of visceral fat levels in T2, T3 and T4 with respect to T1, with the lowest values after surgery (7.4) (Table 3).

All participants lost body weight and body fat percentage during the prehabilitation phase and after surgery but increased slightly at T4. Body weight declined from 78.24 kg (T1) to 76.52 kg (T2) and 73.68 kg (T3), followed by partial recovery to 75.7 kg at T4, while body fat percentage decreased from 22.34% (T1) to 22.28% (T2) and 21.18% (T3), with a slight increase to 21.82% at T4 (Table 3).

As shown in Table 3, only muscle mass of participant 3 increased during the prehabilitation phase but after surgery, all participants showed lower values with respect to baseline. In general, muscle mass decreased from 58.1 kg (T1) to 56.52 kg (T2) and 55.28 kg (T3), with partial recovery to 56.64 kg at T4, although only participant 4 returned to baseline levels (Table 3).

Muscle Strength

**Figure 2:**
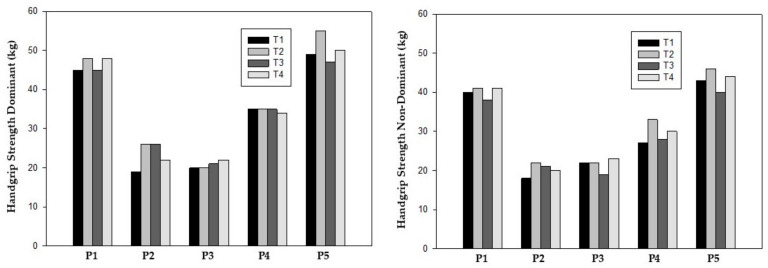
Handgrip strength test.

Handgrip strength improved in the dominant and non-dominant hands in prehabilitation phase, increasing from a mean of 33.6 ± 13.8 kg at T1 to 38.4 ± 15.7 kg at T2 for the dominant hand and 30.0 ± 11.0 kg (T1) to 34.4 ± 12.4 kg (T2) for the non-dominant hand. Considering post-surgery, only participants 2 and 4 increased handgrip strength in both hands. However, at T4, all participants showed higher values compared to baseline except for participant 4 for the dominant hand (Table 4) (Figure 2).

**Figure 3:**
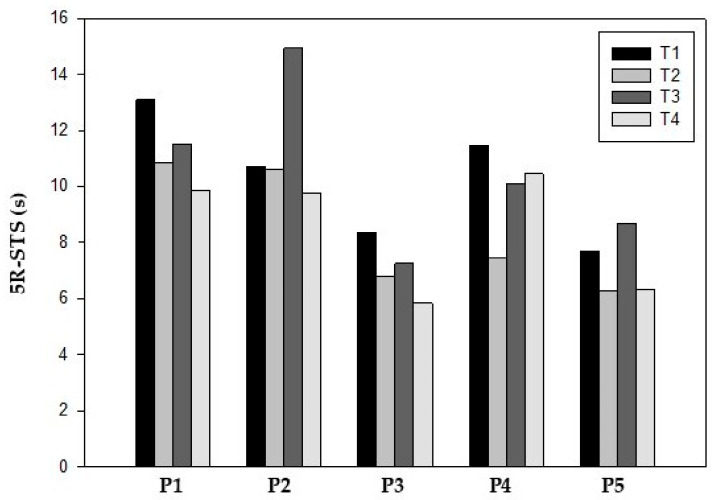
Five-repetition sit-to-stand test.

Lower limb strength, as measured by 5R-STS, also showed functional improvements in all participants at T2. The mean completion time improved from 10.87 ± 2.92 s at T1 to 8.53 ± 2.37 s at T2. After surgery (T3), participants 2 and 5 had the worst values compared to T1 but all participants showed better values at T4 compared to baseline (8.45 ± 2.18 s) (Table 4) (Figure 3).

Psychosocial factors

Psychosocial adaptation varied among participants in the different dimensions (Table 5).

Regarding the Fighting Spirit dimension, all participants remained relatively stable over time except for participant 2, who showed lower values after intervention. Results for the Anxious Preoccupation dimension showed an increase for all participants in T4 and T5. For the Fatalism dimension, participants 2, 3 and 4 increased their scores at T4, but at T5, only participants 2 and 3 maintained their scores. Participants 1, 2 and 4 increased their scores on the Helplessness/Hopelessness dimension at T4, but in follow-up (T5), participants 1 and 2 maintained their scores. In the Cognitive Avoidance dimension, all participants except participant 1 had higher scores at T4 and T5 (Table 5).

Psychological distress, as measured by the hospital anxiety and depression scale (HADS) (Table 6), showed a favorable evolution over the course of the intervention. Although participant 1 increased his score by 1 point, anxiety levels (HADS-A) decreased significantly from 10.2 ± 4.79 at baseline (T1) to 7.2 ± 4.26 at both T4 and T5 (−3.0 points). Similarly, depression scores (HADS-D) decreased from 7.4 ± 3.13 to 6.0 ± 1.22 (−1.4 points), with participants 1 and 2 showing higher values at T4 and T5.

Health-related quality of life (HRQoL)

The EQ-5D index showed minimal differences between the participants along the prehabilitation and post-surgery rehabilitation (Table 7).

During prehabilitation, the total scores of patient’s health state varied from 0.973 (T1) to 0.958 (T2), with participant 2’s pain/discomfort scores worsening. After surgery, all participants’ scores worsened at T3 but improved during postoperative rehabilitation (T4) and remained stable at follow-up (T5).

In contrast, the EQ visual analogue scale (VAS), which captures subjective self-rated health, revealed a more dynamic pattern. Mean VAS scores improved slightly after prehabilitation (T1: 77.0 ± 13.04; T2: 79 ± 10.84), followed by a marked decline post-surgery (T3: 58 ± 8.37). Partial recovery was observed during the post-surgery rehabilitation phase (T4: 68.8 ± 27.80), which continued to a modest extent at follow-up (T5: 67 ± 18.57), although baseline values were not reached (Table 7).

#### Postoperative Complications and Safety Monitoring

No major postoperative complications were reported among the five participants, as defined by the Clavien–Dindo classification of grade III or higher. Two patients experienced minor complications within the first 21 days following surgery: one case of seroma at the surgical site and another of mild localized infection at a suture point. Both events were managed conservatively and did not require reintervention or result in extended hospital stays. No hospital remission occurred during the 30-day postoperative period.

None of the participants received neoadjuvant or adjuvant chemotherapy during the study period. Therefore, the influence of chemotherapy on physical or psychosocial outcomes was not applicable in this cohort.

Throughout the telerehabilitation intervention, no adverse events related to the prescribed exercise program were reported. Safety monitoring was conducted weekly via self-reported forms and asynchronous check-ins through the HEFORA platform. Participants were explicitly instructed to discontinue physical activity and notify the clinical team in case of any concerning symptoms, including pain, fever, dyspnea, general malaise, palpitations, dizziness, or fatigue. The platform’s integrated feedback system facilitated real-time monitoring and prompt response by the physiotherapy team, ensuring patient safety throughout both the prehabilitation and postoperative rehabilitation phases.

## 3. Discussion

This case series evaluated the effects of an asynchronous telerehabilitation program, integrated into both the prehabilitation and postoperative recovery phases, on functional capacity, body composition, muscle strength, psychosocial adjustment, and QoL outcomes in five patients undergoing laparoscopic CRC surgery. The findings suggest that such a digital intervention is not only feasible but could also promote positive trends across multiple clinical dimensions.

During the prehabilitation phase, participants showed an increase in distance walked during the 6MWT, with further significant improvements observed post-rehabilitation assessment. The improvement was above the threshold of 50 m, which is the minimum detectable change (MDC) in patients with similar characteristics to those in our study [26]. These changes align with findings from Beyer et al., who reported a pooled mean increase of 63.47 m (95% CI 28.18–98.76) in postoperative cancer patients undergoing structured exercise interventions [15]. Yang et al. similarly identified moderate-quality evidence supporting improved physical fitness following exercise-based rehabilitation in CRC patients [14].

Muscle strength outcomes showed more modest improvements. Handgrip strength increased in both dominant and non-dominant hands during the prehabilitation phase, with partial maintenance or further enhancement after surgery; however, only one participant exceeded the MDC threshold of 6.5 kg. Similarly, although 5R-STS improved, no participants surpassed the corresponding MDC. The changes in handgrip strength and lower limb performance observed in our study, although very modest, are in line with the results of previous studies on prehabilitation and telerehabilitation interventions. Pesce et al. showed that prehabilitation can significantly improve functional recovery after CCR surgery, with measurable improvements in physical performance parameters [42]. Similarly, Piraux et al. reported that structured preoperative interventions in high-risk surgical patients resulted in modest but clinically relevant improvement muscle strength and overall functional capacity [43]. These subthreshold changes may reflect the short duration of prehabilitation (two weeks), which may not have been sufficient to induce neuromuscular adaptations, consistent with previous research suggesting that longer or more intensive interventions may be required when it comes to preoperative strategies [35,42,43]. These findings emphasize the need to interpret functional performance measures in small samples with caution, particularly when effect sizes are established below thresholds for clinical significance.

Regarding body composition, there was a modest reduction in waist circumference and weight at post-prehabilitation and post-rehabilitation. Muscle mass and body fat percentage also decreased. While these results may seem contradictory, particularly the loss of muscle mass, they are consistent with previous studies indicating that short-term multimodal interventions can lead to body recomposition, depending on baseline nutritional status, adherence, and the balance between aerobic and resistance training components [44,45]. Bojesen et al. highlighted that high-intensity prehabilitation may improve functional outcomes even when changes in body composition are minimal [17], while Carli et al. found that multimodal prehabilitation could modulate lean body mass, especially when combined with nutritional support [46]. The muscle loss observed in some participants could be due to surgical stress, reduced caloric intake or an inadequate anabolic stimulus during the intervention, emphasizing the need for ongoing nutritional monitoring and resistance-focused programming in future iterations [47].

Psychosocial outcomes in this study exhibited a multidimensional profile. On the one hand, general psychological distress, as measured by the HADS, declined during the intervention, suggesting a beneficial impact on overall emotional well-being. On the other hand, subscales of the MAC scale revealed rising levels of cancer-specific distress, particularly anxious preoccupation. This divergence points to a potential disconnect between general mood stabilization and disease-specific coping, consistent with observations from Wu et al. [48]. In a remote context, continuous self-monitoring without the social environment of an on-site clinic may increase physical vigilance and anticipatory anxiety. This response shift phenomenon leads patients to reinterpret their limitations and drive-up MAC scores despite objective functional improvements. The additional burden of self-regulation and social isolation could further weaken self-efficacy and increase distress. These findings underscore the complexity of psychological adaptation in oncologic populations and support the integration of tailored psychological support. Incorporating brief psycho-oncological modules into telerehabilitation sessions may realign the physical and psychological course of recovery [39].

With regard to HRQoL, which was assessed using the EQ-5D index and the EQ-VAS, there was general stability across the time points. The EQ-5D index remained consistently high, suggesting that participants maintained their ability to perform daily activities and experienced no major impairment in functional health [49]. These findings are consistent with those of Downing et al. who reported that, although one-third of colorectal cancer survivors rated their health as perfect on the EQ-5D index, the majority still experienced problems, particularly with pain/discomfort and usual activities, which were also temporarily observed post-surgery [50]. In contrast, the EQ-VAS, which reflects patients’ subjective perception of health, showed greater variability: a slight improvement after prehabilitation, a significant decline after surgery and only partial recovery at follow-up [51]. Nonetheless, these results also highlight the importance of integrating symptom management and psychosocial support within telerehabilitation models to more fully restore perceived QoL [41].

This case series illustrates the practical application of an asynchronous telerehabilitation model in a CCR patient population. Unlike most prehabilitation programs, which are delivered in person or via synchronous online sessions, this approach empowered patients to self-manage their intervention using a digital platform supported by remote monitoring. Despite varying outcomes, all participants successfully completed the program, suggesting that this model offers greater accessibility, particularly for those facing geographic, time or mobility challenges [52].

Although the exploratory nature of this case series limits definitive conclusions regarding clinical efficacy and generalizability, the results provide valuable insight into the evolution of functional, physical, and psychosocial parameters over time in the context of telerehabilitation. The study’s primary limitations include the small sample size, absence of a control group, and the lack of stratification by tumor type, disease stage or comorbidities. These factors likely contributed to the variability observed at baseline and may have influenced individual responses to the intervention. Nonetheless, the observed trends suggest that asynchronous telerehabilitation is not only feasible but also potentially sensitive to detecting meaningful changes in patient outcomes.

Another limitation relates to the restricted duration of the prehabilitation period, which was limited to two weeks because this is the period between diagnosis and surgery in the Spanish healthcare system. While this timeframe reflects real-world clinical conditions, it may not allow for the full realization of physiological adaptations, particularly in terms of muscle strength and functional reserve. Future investigations should explore the utility of extended or personalized prehabilitation timelines based on baseline functional capacity and individual risk profiles.

Additionally, the divergent trajectories observed in psychosocial measures warrant further consideration. This dissociation underscores the complex psychological landscape faced by oncology patients and reinforces the importance of embedding structured psychological support within telerehabilitation frameworks.

Future investigations should focus on the identification of patient-reported barriers and facilitators to engagement, as well as the development of adaptive intervention models that allow for personalization based on baseline characteristics, preferences, or clinical risk. Enhancing digital monitoring capabilities and integrating patient-centered design principles may further improve long-term outcomes.

## 4. Conclusions

This case series explored the integration of an asynchronous telerehabilitation program into the prehabilitation and rehabilitation continuum for patients undergoing laparoscopic colorectal cancer surgery. While some improvements were observed in physical performance, body composition, muscle strength, psychosocial outcomes and health-related quality of life, the small sample size and lack of a control group constrain the generalizability of the findings. These preliminary results should be viewed as exploratory and hypothesis-generating. The asynchronous delivery model, which promotes patient engagement and self-management through remote monitoring, represents a potentially feasible approach and acceptable alternative to conventional rehabilitation strategies. However, given the exploratory nature of these results, further research (including randomized controlled trials with larger and more homogeneous populations) is needed to determine clinical effectiveness, optimize intervention components and evaluate the cost-effectiveness of telerehabilitation within oncology treatment pathways.

## Figures and Tables

**Table 1:** Sociodemographic characteristics.

	Age	Gender	Weight (kg)	Height (cm)	BMI (kg/cm)	Marital Status	Education Level	Employment Status
**Participant 1**	36	Male	92.8	191	25.7	Single	Secondary	Employed
**Participant 2**	62	Female	56.9	166	20.6	Divorced	Primary	Employed
**Participant 3**	52	Female	53.4	164	20.8	Divorced	University	Employed
**Participant 4**	56	Male	95.6	179	29.8	Married	Secondary	Employed
**Participant 5**	62	Male	92.5	179	28.9	Married	University	Employed

BMI = Body Mass Index.

**Table 2:** Submaximal functional capacity.

Variable	Participant	Assessment				Change Score		
		T1	T2	T3	T4	T2–T1	T3–T1	T4–T1
**6MWT** **(meters)**	**P1**	600	690	615	670	90	15	70
	**P2**	570	660	400	540	90	−170	−30
	**P3**	480	720	660	770	240	180	290
	**P4**	550	630	554	630	80	4	80
	**P5**	586	680	514	570	94	−72	−16
	**Mean ± SD**	**557.0 ± 47**	**676.0 ± 33.6**	**548.6 ± 100.2**	**652.5 ± 90.4**	**67.9 ± 118.8**	**130.9 ± 26.6**	**128.0 ± 78.8**

P1, P2…: participant 1, participant 2…; T1: pre-prehabilitation; T2: post-prehabilitation; T3: pre-rehabilitation; T4: post-rehabilitation.

**Table 3:** Body composition.

Variable	Participant	Assessment				Change Score		
		T1	T2	T3	T4	T2–T1	T3–T1	T4–T1
**Waist Circumference (cm)**	**P1**	94	92	90	92	−2	−4	−2
	**P2**	72	72	72	74	0	0	2
	**P3**	75	71	70	73	−4	−5	−2
	**P4**	109	105.5	103	105	−3.5	−6	−4
	**P5**	102	100	98	99	−2	−4	−3
	**Mean ± SD**	**90.4 ± 16.3**	**83.8 ± 16.3**	**86.6 ± 15**	**88.6 ± 14.5**	**(−) 2.3 ± 1.6**	**(−) 3.8 ± 2.3**	**(−) 1.8 ± 2.3**
**Hip Circumference (cm)**	**P1**	106	106	106	106	0	0	0
	**P2**	99	91	92.5	94	−8	−6.5	−5
	**P3**	93	89	92.5	93	−4	−0.5	0
	**P4**	105	104.5	104	104	−0.5	−1	−1
	**P5**	106	105	104	106	−1	−2	0
	**Mean ± SD**	**101.8 ± 5.7**	**97.8 ± 9**	**104.7 ± 1.2**	**100.6 ± 6.5**	**(−) 2.7 ± 3.3**	**(−) 2.0 ± 2.6**	**(−) 1.2 ± 2.2**
**Visceral Fat Level**	**P1**	5	5	5	5	0	0	0
	**P2**	6	6	5	6	0	−1	0
	**P3**	4	4	3	4	0	−1	0
	**P4**	12	13	12	10	1	0	−2
	**P5**	13	14	12	16	1	−1	2
	**Mean ± SD**	**8 ± 4.2**	**8.4 ± 4.7**	**7.4 ± 4.3**	**8.2 ± 4.9**	**0.4 ± 0.5**	**(−) 0.6 ± 0.5**	**0 ± 1.4**
**Weight (kg)**	**P1**	92.8	89.2	86.8	90.6	−3.6	−6	−2.2
	**P2**	56.9	56.1	54.2	55.7	−0.8	−2.7	−1.2
	**P3**	53.4	52.5	50.9	51.4	−0.9	−2.5	−3
	**P4**	95.6	94.5	89.1	90.5	−1.1	−6.5	−5.1
	**P5**	92.5	90.3	87.4	90.3	−2.2	−5.1	−2.2
	**Mean ± SD**	**78.2 ± 22.6**	**76.5 ± 21.8**	**73.7 ± 20.5**	**875.7 ± 21.4**	**(−) 1.7 ± 1.2**	**(−) 4.6 ± 1.9**	**(−) 2.7 ± 1.5**
**Body Fat (Percentage)**	**P1**	14.7	15.1	15	14.8	0.4	0.3	0.1
	**P2**	26.1	26.3	26.5	26.4	0.2	0.4	0.3
	**P3**	23.5	20.5	20.4	23.1	−3	−3.1	−0.4
	**P4**	23.2	24.2	24.1	18.8	1	0.9	−4.4
	**P5**	24.2	25.3	22.9	26	1.1	−1.3	1.8
	**Mean ± SD**	**24.5 ± 1.5**	**22.3 ± 4.6**	**21.8 ± 4.4**	**21.8 ± 5**	**(−) 0.1 ± 1.7**	**(−) 0.6 ± 1.6**	**(−) 0.5 ± 2.3**
**Muscle Mass (Percentage)**	**P1**	75.3	72	70.2	73.4	−3.3	−5.1	−1.9
	**P2**	39.9	39.0	39.4	38.9	−0.9	−0.5	−1
	**P3**	38.7	39.2	38.5	37.6	0.5	−0.2	−1.1
	**P4**	69.9	68.1	64.2	69.8	−1.8	−5.7	−0.1
	**P5**	66.7	64.1	64.1	63.5	−2.6	−2.6	−3.2
	**Mean ± SD**	**58.1 ± 17.4**	**60.9 ± 14.8**	**55.3 ± 15.1**	**56.6 ± 17.2**	**(−) 1.6 ± 1.5**	**(−) 2.8 ± 2.5**	**(−) 1.5 ± 1.2**
**BMI (Body Mass Index)**	**P1**	25.7	24.7	24.3	24.8	−1	−1.4	−0.9
	**P2**	20.6	20.4	19.9	20.2	−0.2	−0.7	−0.4
	**P3**	20.8	20.5	19.9	20.1	−0.3	−0.9	−0.7
	**P4**	29.8	29.5	27.8	28.2	−0.3	−2.3	−1.6
	**P5**	28.9	28.5	27.3	27.8	−0.4	−1.6	−1.1
	**Mean ± SD**	**25.2 ± 4.3**	**24.7 ± 4.3**	**23.8 ± 3.8**	**24.2 ± 3.9**	**(−) 0.4 ± 0.3**	**(−) 1.4 ± 0.6**	**(−) 0.9 ± 0.5**

P1, P2…: participant 1, participant 2…; T1: pre-prehabilitation; T2: post-prehabilitation; T3: pre-rehabilitation; T4: post-rehabilitation.

**Table 4:** Muscle strength.

Variable	Participant	Assessment				Change Score		
		T1	T2	T3	T4	T2–T1	T3–T1	T4–T1
**Handgrip Strength Dominant (kg)**	**P1**	45	48	45	48	3	0	3
	**P2**	19	26	26	22	7	7	3
	**P3**	20	20	21	22	0	1	2
	**P4**	35	35	35	34	0	0	−1
	**P5**	49	55	47	50	6	−2	1
	**Mean ± SD**	**33.6 ± 13.8**	**36.8 ± 14.7**	**34.8 ± 11.4**	**35.2 ± 13.5**	**3.2 ± 3.3**	**1.2 ± 3.4**	**1.6 ± 1.7**
**Handgrip Strength Non-dominant (kg)**	**P1**	40	41	38	41	1	−2	1
	**P2**	18	22	21	20	4	3	2
	**P3**	22	22	19	23	0	−3	1
	**P4**	27	33	28	30	5	1	3
	**P5**	43	46	40	44	3	−3	1
	**Mean ± SD**	**30.0 ± 11**	**32.8 ± 10.9**	**29.2 ± 9.6**	**31.6 ± 10.6**	**2.6 ± 2.1**	**(−) 0.8 ± 2.7**	**1.6 ± 0.9**
**Sit-to-stand** **5R-STS (s)**	**P1**	13.09	10.84	11.5	9.84	−2.25	−1.59	−3.25
	**P2**	10.72	10.63	14.94	9.78	−0.09	4.22	−0.94
	**P3**	8.37	6.8	7.25	5.85	−1.57	−1.12	−2.52
	**P4**	11.46	7.46	10.1	10.46	−4	−1.36	−1
	**P5**	7.69	6.28	8.69	6.34	−1.41	1	−1.35
	**Mean ± SD**	**10.3 ± 2.2**	**7.8 ± 2**	**10.5 ± 2.9**	**8.5 ± 2.2**	**1.9 ± 1.4**	**0.2 ± 2.5**	**(−) 1.8 ± 1**

P1, P2…: participant 1, participant 2…; T1: pre-prehabilitation; T2: post-prehabilitation; T3: pre-rehabilitation; T4: post-rehabilitation.

**Table 5:** Psychosocial factors: The mental adjustment to cancer scale (MAC).

Variable	Participant	Assessment			Change Score	
		T1	T4	T5	T4–T1	T5–T1
**Fighting Spirit (FS)**	**P1**	30	31	31	1	1
	**P2**	34	28	28	−6	−6
	**P3**	24	26	26	−2	−2
	**P4**	29	31	29	2	0
	**P5**	29	28	28	−1	−1
	**Mean ± SD**	**29.2 ± 3.6**	**28.8 ± 2.2**	**28.4 ± 1.8**	**(−) 1.0 ± 3.6**	**(−) 1.5 ± 3.1**
**Anxious Preoccupation (AP)**	**P1**	19	21	21	2	2
	**P2**	19	22	22	3	3
	**P3**	19	23	23	4	4
	**P4**	16	21	20	5	4
	**P5**	19	26	26	7	7
	**Mean ± SD**	**18.4 ± 1.3**	**22.6 ± 2.1**	**22.4 ± 2.3**	**4.2 ± 1.9**	**4.0 ± 1.9**
**Fatalism (FA)**	**P1**	24	16	16	−8	−8
	**P2**	17	18	18	1	1
	**P3**	11	13	13	2	2
	**P4**	15	17	14	2	−1
	**P5**	19	15	15	−4	−4
	**Mean ± SD**	**17.2 ± 4.8**	**15.8 ± 1.9**	**15.2 ± 1.9**	**(−) 1.4 ± 4.4**	**(−) 2.0 ± 4.1**
**Helplessness/Hopelessness (HH)**	**P1**	22	25	25	3	3
	**P2**	22	24	24	2	2
	**P3**	30	27	27	−3	−3
	**P4**	23	27	23	4	0
	**P5**	26	25	25	−1	−1
	**Mean ± SD**	**24.6 ± 3.4**	**25.6 ± 1.3**	**24.8 ± 1.5**	**1.0 ± 2.9**	**0.2 ± 2.4**
**Cognitive Avoidance (CA)**	**P1**	8	7	7	−1	−1
	**P2**	5	7	7	2	2
	**P3**	5	7	7	2	2
	**P4**	6	7	7	1	1
	**P5**	6	7	7	1	1
	**Mean ± SD**	**6.0 ± 1.2**	**7.0 ± 0**	**7.0 ± 0**	**1.0 ± 1.2**	**1.0 ± 1.2**

P1, P2…: participant 1, participant 2…; T1: pre-prehabilitation; T2: post-prehabilitation; T3: pre-rehabilitation; T4: post-rehabilitation; T5: follow-up.

**Table 6:** Psychosocial factors: Hospital anxiety and depression scale (HADS).

Variable	Participant	Assessment			Change Score	
		T1	T4	T5	T4–T1	T5–T1
**HADS-A**	**P1**	4	5	5	1	1
	**P2**	13	9	9	−4	−4
	**P3**	14	13	13	−1	−1
	**P4**	5	3	3	−2	−2
	**P5**	15	6	6	−9	−9
	**Mean ± SD**	**10.2 ± 5.3**	**7.2 ± 3.9**	**7.2 ± 3.9**	**(−) 3.0 ± 3.8**	**(−) 3.0 ± 3.8**
**HADS-D**	**P1**	6	7	7	1	1
	**P2**	4	5	5	1	1
	**P3**	12	7	7	−5	−5
	**P4**	5	5	5	0	0
	**P5**	10	6	6	−4	−4
	**Mean ± SD**	**7.4 ± 3.4**	**6.0 ± 1**	**6.0 ± 1**	**(−) 1.4 ± 2.9**	**(−) 1.4 ± 2.9**

P1, P2…: participant 1, participant 2…; T1: pre-prehabilitation; T4: post-rehabilitation; T5: follow-up.

**Table 7:** Quality of life: EuroQol-5D.

**T1**							
**Participant**	**Mobility**	**Self-Care**	**Usual Activities**	**Pain/Discomfort**	**Anxiety/Depression**	**EQ-5D Index**	**EQ-VAS**
**P1**	1	1	1	1	1	1.000	60
**P2**	1	1	1	1	1	1.000	80
**P3**	1	1	1	1	2	0.932	70
**P4**	1	1	1	1	1	1.000	80
**P5**	1	1	1	1	2	0.932	95
**Mean ± SD**						**0.973 ± 0.04**	**77.0 ± 13.04**
**T2**							
**Participant**	**Mobility**	**Self-Care**	**Usual Activities**	**Pain/Discomfort**	**Anxiety/Depression**	**EQ-5D Index**	**EQ-VAS**
**P1**	1	1	1	2	1	0.924	65
**P2**	1	1	1	1	1	1.000	80
**P3**	1	1	1	1	2	0.932	75
**P4**	1	1	1	1	1	1.000	80
**P5**	1	1	1	1	2	0.932	95
**Mean ± SD**						**0.958 ± 0.04**	**79.0 ± 10.84**
**T3**							
**Participant**	**Mobility**	**Self-Care**	**Usual Activities**	**Pain/Discomfort**	**Anxiety/Depression**	**EQ-5D Index**	**EQ-VAS**
**P1**	1	1	1	2	1	0.924	60
**P2**	1	1	2	2	1	0.849	50
**P3**	1	1	1	1	2	0.932	50
**P4**	1	1	1	2	2	0.857	70
**P5**	1	1	1	1	1	1.000	60
**Mean ± SD**						**0.912 ± 0.06**	**58.0 ± 8.37**
**T4**							
**Participant**	**Mobility**	**Self-Care**	**Usual Activities**	**Pain/Discomfort**	**Anxiety/Depression**	**EQ-5D Index**	**EQ-VAS**
**P1**	1	1	1	1	1	1.000	40
**P2**	1	1	1	1	2	0.932	50
**P3**	1	1	1	1	2	0.932	40
**P4**	1	1	1	2	2	0.857	90
**P5**	1	1	1	1	1	1.000	95
**Mean ± SD**						**0.944 ± 0.06**	**68.8 ± 27.80**
**T5**							
**Participant**	**Mobility**	**Self-Care**	**Usual Activities**	**Pain/Discomfort**	**Anxiety/Depression**	**EQ-5D Index**	**EQ-VAS**
**P1**	1	1	1	1	1	1.000	50
**P2**	1	1	1	1	2	0.932	70
**P3**	1	1	1	1	2	0.932	50
**P4**	1	1	1	2	2	0.857	70
**P5**	1	1	1	1	1	1.000	95
**Mean ± SD**						**0.944 ± 0.06**	**67.0 ± 18.57**

P1, P2…: participant 1, participant 2…; T1: pre-prehabilitation; T2: post-prehabilitation; T3: pre-rehabilitation; T4: post-rehabilitation; T5: follow-up.

## Data Availability

The data supporting the findings of this study have been provided for review. Due to privacy and protection concerns, the data cannot be made publicly accessible. This measure ensures the confidentiality and security of sensitive information. For this reason, the raw data will be available upon request from the corresponding author.

## Abbreviations

The following abbreviations are used in this manuscript:

CRC	Colorectal Cancer
WHO	World Health Organization
QoL	Quality of Life
ERAS	Enhanced Recovery After Surgery
ASA	American Society of Anaesthesiologists
BMI	Body Mass Index
6MWT	Six-Minute Walk Test
ATS	American Thoracic Society
ERS	European Respiratory Society
BIA	Bioelectrical Impedance Analysis
HGS	Handgrip Strength Test
5R-STS	Five-Repetition Sit-to-Stand Test
MAC	Mental Adjustment to Cancer
HADS	Hospital Anxiety and Depression Scale
HRQoL	Health-Related Quality of Life
EQ-5D	EuroQol-5D
EQ-VAS	Visual Analogue Scale
T1	Pre-prehabilitation
T2	Post-prehabilitation
T3	Pre-rehabilitation
T4	Post-rehabilitation
T5	Follow-up
FS	Fighting Spirit
AP	Anxious Preoccupation
FA	Fatalism
HH	Helplessness/Hopelessness
CA	Cognitive Avoidance
MDC	Minimum Detectable Change
